# Humoral Immune Responses to *Pneumocystis jirovecii* Antigens in HIV-Infected and Uninfected Young Children with *Pneumocystis* Pneumonia

**DOI:** 10.1371/journal.pone.0082783

**Published:** 2013-12-26

**Authors:** Kpandja Djawe, Kieran R. Daly, Linda Levin, Heather J. Zar, Peter D. Walzer

**Affiliations:** 1 Department of Environmental Health, University of Cincinnati College of Medicine, Cincinnati, Ohio, United States of America; 2 Department of Internal Medicine, University of Cincinnati College of Medicine, Cincinnati, Ohio, United States of America; 3 Research Service, Veterans Affairs Medical Center, Cincinnati, Ohio, United States of America; 4 Department of Pediatrics and Child Health, University of Cape Town, Cape Town, South Africa; Albert Einstein College of Medicine, United States of America

## Abstract

**Background:**

Humoral immune responses in human immunodeficiency virus (HIV)-infected and uninfected children with *Pneumocystis* pneumonia (PcP) are poorly understood.

**Methods:**

Consecutive children hospitalized with acute pneumonia, tachypnea, and hypoxia in South Africa were investigated for PcP, which was diagnosed by real-time polymerase chain reaction on lower respiratory tract specimens. Serum antibody responses to recombinant fragments of the carboxyl terminus of *Pneumocystis jirovecii* major surface glycoprotein (MsgC) were analyzed.

**Results:**

149 children were enrolled of whom 96 (64%) were HIV-infected. PcP occurred in 69 (72%) of HIV-infected and 14 (26%) of HIV-uninfected children. HIV-infected children with PcP had significantly decreased IgG antibodies to MsgC compared to HIV-infected patients without PcP, but had similar IgM antibodies. In contrast, HIV-uninfected children with PcP showed no change in IgG antibodies to MsgC, but had significantly increased IgM antibodies compared to HIV-uninfected children without PCP. Age was an independent predictor of high IgG antibodies, whereas PcP was a predictor of low IgG antibodies and high IgM antibodies. IgG and IgM antibody levels to the most closely related MsgC fragments were predictors of survival from PcP.

**Conclusions:**

Young HIV-infected children with PcP have significantly impaired humoral immune responses to MsgC, whereas HIV-uninfected children with PcP can develop active humoral immune responses. The children also exhibit a complex relationship between specific host factors and antibody levels to MsgC fragments that may be related to survival from PcP.

## Introduction

*Pneumocystis jirovecii* is an opportunistic pulmonary pathogen of worldwide distribution. Primary *P. jirovecii* infection is acquired during the first few months of life, and is either asymptomatic or a self-limited infection [1,2]. Seroepidemiological studies have shown that by 2-3 years of age, most healthy children have been infected with the organism [2-6]. *P. jirovecii* remains a major cause of life-threatening pneumonia (termed “PcP”) in children who are immunocompromised by human immunodeficiency virus (HIV) infection, cancer, or other disorders. This is especially true in children in low or middle income countries where the disease occurs in 10% to 49% of HIV-infected children hospitalized for pneumonia with an in-hospital mortality rate of 20% to 63% [7-11]. 

The diagnosis of PcP has traditionally been made by the demonstration of the organism by histologic or immunofluorescent staining in specimens that have been carefully obtained from the respiratory tract. It is likely that this method underestimates the true incidence of PcP, particularly in areas with limited laboratory facilities [1-14]. Recent studies in adults with and without PcP have shown that the polymerase chain reaction (PCR), particularly real time (RT)-PCR, is more sensitive than microscopy in detecting *P. jirovecii* and may also distinguish colonization from active disease [15-21]. We have obtained similar results with the use of RT-PCR in the diagnosis of PcP and tuberculosis in young children [22-24]. 

HIV-infected children with PcP have markedly decreased CD4+ cell counts and broad defects in cellular and humoral function, as illustrated by their low serum antibody levels to common infectious agents and to immunizations [25-28]. HIV-uninfected children, who are exposed to HIV but remain HIV-negative, have been reported to be at greater risk for developing PcP than HIV-uninfected, unexposed children [29,30]; however, the reasons for the difference are unclear [31-34].

Little is known about the role of specific immune responses to *P.jirovecii* in HIV-infected children with or without PcP. Over the past decade, the development of recombinant antigens has begun to change this picture. The major surface glycoprotein (Msg) of *P.jirovecii* plays a central role in the interaction of the organism with the host; contains protective B and T cell epitopes; is encoded by a multi-gene family, and is capable of antigenic variation [35-39]. We have developed 3 recombinant fragments (MsgA, MsgB, MsgC1) that span the length of a single Msg isoform [23], and analyzed their reactivity in both adult and pediatric populations [6,40-48]. MsgC1, which contains the carboxyl terminus and is the most conserved part of Msg, showed the most promise; thus, 3 variants (MsgC3, MsgC8, and MsgC9) were developed to better characterize the reactivity of the antibodies [40-48].

The aims of this study were: 1) to characterize the IgG and IgM antibody responses to MsgC fragments in HIV-infected and HIV-uninfected children hospitalized with PcP (PcP+) and other causes of pneumonia (PcP-); 2) to identify specific host factors that are independent predictors of these antibody levels; 3) to determine if any of the antibody responses are independent predictors of mortality from PcP.

## Materials and Methods

### Study Design

A prospective study was conducted of consecutive children admitted to the Red Cross War Memorial Children’s Hospital, Cape Town, South Africa, with hypoxic pneumonia from Nov 2006 to Aug 2008 [22]. Clinical criteria for suspected PCP were an acute onset (<2 weeks) of a respiratory illness; the presence of age-specific tachypnea and hypoxia (arterial oxygen saturation <92% in room-air); bilateral lung disease (not associated with wheezing); and the presence of a risk factor for PCP (e.g. HIV-infection, malnutrition, immunosuppressive therapy). These criteria were established to ensure that subjects were seriously ill with pneumonia and had significant risk factors for PCP. Initial specimens were obtained within the first 48 hours of admission. Exclusion criteria included treatment of PcP in the preceding 2 weeks or treatment for PCP for the current admission for more than 48 hours. 

Blood specimens were collected at enrollment for HIV testing (if status was unknown), CD4 cell measurement, and a serum specimen was frozen for further analysis. A child was defined as HIV-infected if he/she had a positive HIV PCR (Roche) and was younger than 18 months or a positive HIV ELISA for antibodies (Abbott) in older children. HIV exposure was defined as HIV-seropositive by ELISA and negative by HIV PCR. 

Upper respiratory tract (URT) specimens were obtained from nasopharyngeal aspirates (NPAs) and lower respiratory tract (LRT) specimens by induced sputum (IS) or bronchoalveolar lavage (BAL) in a standard manner within 48 hours of admission. The specimens were examined for the presence of *P. jirovecii* by RT-PCR and microscopic techniques (direct immunofluorescence (IF) using a monoclonal antibody, silver staining) and other organisms as described (23). The data showed that RT-PCR was far more sensitive than microscopic techniques in detecting *P. jirovecii* with good specificity. Other organisms that were found included viruses (e.g. cytomegalovirus (CMV), respiratory viruses) and bacteria (e.g. *Staphylococcus aureus, Mycobacterium tuberculosis*). 

 Treatment of PcP included trimethoprim-sulfamethoxazole (TMP-SMX) and corticosteroids; other antimicrobial drugs, or antiretroviral drugs were at the discretion of the treating physician. The overall in-hospital mortality rate was 25%, and the case-fatality rate was also significantly higher in PcP patients (40%) than in non-PcP patients (21%). Written informed consent was obtained from a parent or legal guardian. Ethical approval of the study was obtained from the Research and Ethics Committee of the Faculty of Health Sciences, University of Cape Town and the University of Cincinnati Institutional Review Board

### Recombinant Antigens

The DNA fragments containing genes encoding recombinant MsgC1, C3, C8, and C9 fragments were prepared via PCR using DNA isolated from *P. jirovecii*- infected lung or cloned *msg* genes as templates as described [40-42]. Each of the 4 MsgC fragments included approximately 425 amino acids. The fragments exhibited 83 to 99% homology at the nucleotide level and 77 to 99% homology at the putative amino acid level [41]. MsgC3 and MsgC9 were the most closely related fragments with just 3 amino acid substitutions. 

### ELISA

The IgG ELISA was performed as previously described [40-42]. Serum specimens to be analyzed and the standard reference serum were tested against each MsgC fragment. The standard specimens were obtained by testing banks of sera from blood donors and HIV-infected patients. The standard serum for each antigen consisted of a pool of 4 to 6 serum specimens with high antibody reactivity to that antigen. The standard serum was defined as having a value of 100 U in 100 µl of a 1:100 dilution. We used the same standard pools throughout the study, and as a further measure to ensure consistency between assays, we titrated subsequent standard pools against those initial standards. From the standard pool, we generated a standard curve for each Msg construct on each day the assay was used. We used this curve to calculate the units of reactivity to the Msg construct. We diluted test serum samples at 1:100 to 1:200 to fit the linear portion of the curves. Taking into account the dilution, we then calculated units of reactivity [40-42].

 IgM antibodies were analyzed in a similar manner except that an anti-IgM (µ-specific) antibody was used [5]. Variations in the assay results were analyzed using a control serum and were measured for MsgC1 on a per-plate, daily, and 4 day basis; the coefficients of variance (CV) were 3.6 to 7%, 4.8 to 7.4%, and 13.3%, respectively [41]. 

### Data Analysis and Statistics

In the first part of the analysis, we characterized the children as follows: HIV-infected with PcP (PcP+), HIV-infected with other causes of pneumonia (PcP-), HIV-uninfected with PcP (HIV-uninfected/PcP+), and HIV-uninfected without PcP (HIV-uninfected/PcP-). We used medians (interquartile range) or counts (percent) to describe continuous and discrete characteristics, respectively. In the second part of the analysis, quantile regression analysis was employed to compare antibody levels among groups and to determine independent risk factors associated with antibody levels. In our previous publications, means of log-transformed antibody responses to Msg fragments were modeled using Tobit regression, as the transformed uncensored responses approximated a truncated lognormal distribution [6,41-45]. In the present study, however, although some subjects had antibody levels below the limit of detection and the values were censored to “1’, we chose to use Quantile regression. This strategy was primarily due to the large number of extremely high antibody responses which precluded modeling the mean response, even after log-transformation. Quantile regression is a non-parametric method which models the relation between a set of independent variables and conditional quantiles (percentiles) of the dependent variable [49]. Quantile regression has also been used to analyze immunological data with high frequency of undetectable results or “non-detects” [50]. Eilers et al. showed that quantile regression permitted groups to be compared and meaningful linear trends could be computed even though more than 50% of the data was composed of non-detects. In the present study, 29% of antibody responses to MsgC1, 32% to MsgC3, 16% to MsgC8, and 19% to MsgC9 were censored. 

Since non-censored antibody levels in the present study were in the upper quartiles, we compared the groups in the 75th and 90th quantiles, and then determined the factors associated with these conditional percentiles. In the final stage of the analysis, we determined predictors of PCP-related mortality using Cox proportional hazard models. The analysis included HIV+ children with PCP and HIV- children with PCP. SAS for Windows, version 9.2 (SAS Institute, Cary, NC) was used to carry out all statistical analyses, and a 5% significance level was assumed, unless stated otherwise. 

## Results

### Demographic and Clinical Characteristics

Of the 202 children originally enrolled (17), 149 (73%) were included in the present study: 96 (64%) were HIV-infected of whom 69 (72%) had PcP+ (Table 1). These children differed significantly from the HIV-infected/PcP- children in their younger age; lower proportion receiving PcP prophylaxis; greater level of immunosuppression as measured by CD4+ cell count; and greater lung damage as evidenced by higher LDH levels. There was also a trend towards higher mortality. 

**Table 1:** Descriptive Characteristics of the Children at Enrollment.

	**HIV+ (N = 96)**		**HIV- (N=53)**		**All (N=149**	
**Characteristics**	PcP+ (n=69)	PcP- (n=27)	PcP+ (n=14)	PcP- (n=39)	HIV+ (n=96)	HIV- (n=53)
Age (months)	**3.4 (2.7-4.0)**	**5.3 (1.9-10.0)**	3.0 (2.5-3.6)	2.2 (1.2-6.9)	**3.5 (2.7-4.7)**	**2.5 (1.3-4.1)**
Male	23 (33)	14 (52)	9 (64)	19 (49)	37 (39)	28 (53)
CD4 (cells/µl)	**296 (118-590)**	**546 (263-934)**			309 (129-709)	
HIV RNA (x10^6^ copies/mL)	2.9 (0.58-3.0)	2.5 (0.79-3.0)			2.6 (0.58 -3.0)	
ART	42 (55)	15 (33)			57 (59)*	
Breast Feeding	35 (53)	14 (61)	8 (57)	19 (52)	49 (55)	27 (54)
Intubation	1 (4)	1 (1)	1(7)	3 (8)	2 (2)	4 (8)
PcP Prophylaxis	**9 (13)**	**10 (34)**	1 (7)	3 (8)	**19 (20)**	**4 (8)**
LDH (U/L)	**729 (556-1152)**	**532 (290-686)**	487 (432-675)	353 (236-418)	**671 (527-1028)**	**377 (236-610)**
Weight (kg) -for-Age Z Score	4.4 (3.6-5.2)	5.6 (4.0-6.2)	3.6 (3.2-4.5)	4.3 (2.9-6.8)	4.6 (3.7-5.5)	3.9 (3.1-4.9)
Mortality	24 (35)	7 (26)	0 (0)	5 (13)	**31 (32)**	**5 (9)**

Continuous and Discrete Data Were Expressed as Median (IQR) and Count (% N), Respectively.

Note: Bolded values are significantly different. * Proportion of HIV-infected patients on ART = antiretroviral therapy. LDH = serum lactate dehydrogenase. HIV+ = HIV-infected. HIV- = HIV-uninfected

Of the 53 HIV-uninfected patients, 21 (40%) were HIV-exposed. Of the exposed patients, only 3 (14%) had PcP, whereas 11 (34%) of the 32 unexposed patients had PcP. There were no significant differences in demographic or clinical characteristics between HIV-uninfected/PcP+ and HIV-uninfected/PcP- children (Table 1). 

### Serum IgG and IgM Antibody Levels

HIV-infected/PcP+ patients had significantly lower IgG antibody levels than HIV-infected/PcP- patients to MsgC1 in the 90^th^ percentile; to MsgC3 in the 75^th^ and 90^th^ percentiles; and to MsgC8 in the 90^th^ percentile (Table 2). In contrast, no significant differences were seen in IgG antibody levels to the MsgC fragments between HIV-uninfected/PcP+ and HIV-uninfected/PcP- children or between HIV-infected/PcP+ and HIV-uninfected/PcP+ children (Table 2). There were also no significant differences in IgG antibody levels between all HIV-infected and all HIV-uninfected children (data not shown).

**Table 2:** Estimated 75^th^ and 90^th^ Percentiles [SE] of IgG Antibody Responses to MsgC Fragments by PcP Diagnosis among HIV+ and HIV- Children at Enrolment.

		**HIV+**		**HIV-**	
Antigens	Percentile	PcP+ (N=69)	PcP- (N=27)	PcP+ (N=14)	PcP- (N=39)
MsgC1	75^th^	1.00 [0.0]	76.78 [16.0]	3.35 [17.2]	14.35 [12.0]
MsgC1	90^th^	3.88*[6.4]	121.59* [17.0]	23.97 [46.3]	76.01[25.2]
MsgC3	75^th^	1.00*[2.3]	174.27*[62.2]	24.27 [48.9]	4.37 [2.8]
MsgC3	90^th^	18.53* [18.0]	250.90* [55.6]	141.26 [72.7]	32.14 [27.2]
MsgC8	75^th^	1.00 [0.0]	21.77 [49.1]	3.85 [20.3]	1.00 [0.4]
MsgC8	90^th^	1.00* [8.0]	287.47* [130.8]	36.25 [53.0]	4.77 [16.0]
MsgC9	75^th^	1.00 [0.3]	3.10 [31.9]	16.10 [55.2]	1.00 [2.5]
MsgC9	90^th^	8.86 [10.9]	181.03 [134.9]	153.89 [79.7]	13.27 [12.9]

^*^ The groups are significantly different. SE = Standard error. HIV+ = HIV-infected. HIV- = HIV-uninfected

No significant differences were found in IgM antibody levels to any of the MsgC constructs among HIV-infected/PcP+ or HIV-infected/PcP- subjects (Table 3). By contrast, HIV-uninfected/PcP+ patients had significantly higher IgM antibody levels than HIV-uninfected/PcP- patients to MsgC1, C8, and C9 in the 75^th^ percentile (Table 3). We were unable to compare antibody levels between HIV-uninfected, exposed, PcP+ and PcP- children due to the small number (3) of PcP+ children. 

**Table 3:** Estimated 75^th^ and 90^th^ Percentiles [SE] of IgM Antibody Responses to MsgC Fragments by PcP Diagnosis among HIV+ and HIV- Children at Enrolment.

		**HIV+**		**HIV-**	
Antigens	Percentile	PcP+ (N=69)	PcP- (N=27)	PcP+ (N=14)	PcP- (N=39)
MsgC1	75^th^	49.54 [10.7]	50.97 [42.3]	109.64 [41.0]	20.66 [9.0]
MsgC1	90^th^	100.72 [14.0]	202.93 [91.3]	126.94 [19.0]	88.60 [41.1]
MsgC3	75^th^	10.76 [3.6]	9.55 [2.7]	13.21 [10.1]	1.48 [1.8]
MsgC3	90^th^	27.65 [5.6]	18.62 [6.9]	36.15 [9.7]	7.66 [2.6]
MsgC8	75^th^	10.84 [3.8]	9.70 [4.1]	18.27 [9.7]	1.21 [0.9]
MsgC8	90^th^	27.65 [4.2]	15.80 [12.3]	33.02 [9.9]	5.37 [6.3]
MsgC9	75^th^	6.41 [2.2]	5.60 [1.6]	18.72* [11.3]	4.06* [1.8]
MsgC9	90^th^	17.73 [3.3]	10.02 [2.4]	25.20 [31.7]	8.14 [3.5]

^*^ The groups are significantly different. SE = Standard error. HIV+ = HIV-infected. HIV- = HIV-uninfected SE:

### Predictors of IgG and IgM Antibody Responses

Regression analyses were performed to find correlations between host factors and antibody responses. Of the IgG antibody responses, age was a predictor of high antibody levels to MsgC1 in the 75^th^ percentile and PcP was a predictor of low antibody levels to MsgC1 in the 75^th^ and the 90^th^ percentiles (Table 4). Age was also a predictor of high antibody levels to MsgC3, MsgC8, and MsgC9 in the 75^th^ percentiles. Of the IgM antibody responses, PcP was a predictor of high antibody levels to MsgC3 in the 75^th^ and 90^th^ percentiles, and to MsgC9 in the 90^th^ percentile (Table 5).

**Table 4:** Predictors of IgG Antibody Responses to MsgC Fragments.

		75^th^ Percentile		90^th^ Percentile	
Antigens	Characteristics	Estimate	p-value	Estimate	p-value
MsgC1	Age	1.35	<0.01	0.35	0.71
	PcP	-2.83	<0.01	-2.66	<0.01
	HIV	0.00	1.00	-0.19	0.80
MsgC3	Age	4.08	<0.01	1.90	0.19
	PcP	-0.81	0.33	-0.65	0.43
	HIV	-0.95	0.25	-0.61	0.51
MsgC8	Age	3.04	<0.01	3.47	0.13
	PcP	-0.00	1.00	-0.91	0.45
	HIV	-0.00	1.00	-1.35	0.31
MsgC9	Age	2.11	0.03	1.76	0.48
	PcP	0.00	1.00	-0.08	0.95
	PcP Prophylaxis	-0.00	1.00	-0.32	0.82

**Table 5:** Predictors of IgM Antibody Responses to MsgC Fragments.

		75^th^ Percentile		90^th^ Percentile	
Antigens	Characteristics	Estimate	p-value	Estimate	p-value
MsgC1	Age	-0.37	0.38	-0.89	0.06
	PcP	0.49	0.29	-0.41	0.15
	HIV	0.37	0.26	-0.05	0.86
MsgC3	Age	0.71	0.25	0.90	0.12
	PcP	1.16	0.02	1.19	<0.01
	HIV	0.82	0.14	-0.01	0.99
MsgC8	Age	-0.41	0.57	-0.26	0.70
	PcP	1.08	0.06	0.67	0.06
	HIV	0.70	0.21	0.01	0.97
MsgC9	Age	0.25	0.66	0.13	0.82
	PcP	0.74	0.08	0.84	<0.01
	PcP Prophylaxis	-0.26	0.54	-0.20	0.50

### IgG and IgM Antibody Responses as Predictors of PcP-related Mortality

Antibody levels to each MsgC construct, weight, and age were analyzed as predictors of PcP-related mortality using HIV+ with PcP and HIV- with PcP and the results were expressed as the Adjusted Mortality Risk Ratios (RR). Of the IgG antibodies, only the antibody level to MsgC3 approached significance: RR 0.58, 95%CI [0.34-1.00] p= 0.05. Of the IgM antibodies, only the antibody level to MsgC9 was significant: RR 0.64, 95% CI [0.41-0.99] p< 0.05. Thus, the IgG antibody level to MsgC3 and IgM antibody level to MsgC9 were associated with a 36.7% and 39% decrease in PcP-related mortality, respectively. In the sensitivity analysis using only HIV+ children with PCP, the effect of CD4+ cell count on mortality was not statistically significant.

## Discussion

 This study has shown that hospitalized HIV-infected children with PcP (PcP+) had significantly lower serum IgG antibodies to recombinant MsgC fragments than HIV-infected children with pneumonia due to other causes (PcP-). By contrast, HIV- uninfected PcP+ children had significantly higher IgM antibodies to these fragments than HIV-uninfected PcP – patients. Age was a predictor of high IgG antibodies, whereas PcP was a predictor of low IgG antibodies but high IgM antibodies. IgG antibody levels to MsgC3 and IgM antibody levels to MsgC9 also were associated with a reduced mortality from PcP.

 Studies of the humoral and cellular immune responses to a specific respiratory pathogen in any patient with pneumonia depend on accurate etiological diagnosis. Recent reports showing that RT-PCR is more sensitive than microscopic analysis in diagnosing PcP in adults [15-21] and children [23] are important because they increase the number of subjects available for analysis and can also distinguish *P. jiroveci* colonization from disease. HIV-infected children not only have broad immune defects ranging from low CD4 counts to B-cell dysregulation with hyperimmunoglobulinemia, but also have poor antibody responses to specific infectious agents or immunizations [25-28,51,52]. Impaired placental transfer of antibodies from HIV-infected mothers is an important contributor to these poor specific antibody responses, as has been shown with viral, bacterial, and parasitic antigens [53,54]. Since maternal antibodies are the main source of protective humoral immune responses during the first 6 months of life, it is not surprising that many of these infections occur during this time.

 Our HIV-infected/PcP+ children exhibited many of the characteristics described above, including low CD4+ cell counts, development of PcP at age 3-4 months, severe disease, elevated LDH levels, low fraction of patients receiving chemoprophylaxis, and a high mortality rate. These children also had very low serum IgG antibody levels to the MsgC fragments, and were unable to develop an active IgM antibody responses that could distinguish them from HIV-infected/PcP- children. 

In contrast to these young HIV-infected children, adult HIV-infected patients with active PcP or a previous episode of PcP whom we have studied display higher antibody levels to the MsgC fragments than HIV-infected patients who never had PcP or healthy adults [43]. HIV-infected adults hospitalized with active PcP had significantly higher levels of IgM and IgG antibody levels than HIV-infected adults hospitalized with other causes of pneumonia at the time of diagnosis, and the differences in antibody levels were maintained until 3-4 weeks later [45]. The positive predictive values (PPV) for IgG and IgM antibody levels rose from 71.5% and 79.3% at admission to 100% and 89.8%, respectively, at 3-4 weeks. It is likely that most HIV-infected adults have fully developed immune systems and repeated exposures to *P. jirovecii* throughout their lives before they develop PcP, whereas young HIV-infected children have immature immune systems and lesser cumulative exposure to *P. jirovecii*.

The MsgC fragments used in the present and previous reports exhibited a high degree homology, and thus have shared as well as unique antigenic determinants. If one MsgC fragment elicits a good antibody response, other fragments also usually elicit good responses; on the other hand, the MsgC fragments react independently and also have unique epitopes (Refs 41-43). Our previous studies in HIV-infected adults have identified specific host and environmental factors that are independent predictors of antibody levels to one or more of the MsgC fragments [42-44]. Thus, current PcP, previous episode of PcP, age, failure to take PcP chemoprophylaxis, and geographic location have been associated with increased IgG and /or IgM antibody levels, whereas smoking and high LDH levels have been associated with decreased IgG and/or IgM antibody levels [6,43-45].

The present study showed age was independently associated with increased IgG antibody levels, but not to IgM antibody levels. It is likely that similar to antibody responses to other commonly encountered organisms, IgG antibody levels increase on repeated exposure to *P. jirovecii* antigens, as children grow and mature. On the other hand, PcP was associated with low IgG antibody levels to MsgC1, which probably reflects the decreased IgG antibody levels that are maternally derived in the HIV-infected/PcP+ patients. The association of PcP with high IgM antibody levels to MsgC3 and MsgC9 probably reflects the antibody response to PcP in the HIV-uninfected/PcP+ children and the fact that MsgC3 and MsgC9 are closely related.

HIV-uninfected exposed children in the present study had similar, though less severe immune defects (including antibody responses to specific antigens) than HIV-infected children [31-34]. Low antibody levels in HIV-infected mothers and decreased placental transmission of antibodies in HIV-infected exposed infants have played an important role. Yet, when tested at age 16 months following vaccination, antibody responses in HIV- uninfected exposed children were as high as or higher than those in the unexposed children [34].

Our HIV-uninfected/PcP+ children had serum IgG antibody levels to the MsgC fragments that were similar to the antibody levels in HIV-uninfected/PcP- patients. However, the HIV-uninfected/PcP+ children exhibited significantly greater IgM antibody responses to the MsgC fragments than the HIV-uninfected/PcP- children. This finding suggests that HIV-uninfected children can develop an active antibody response to PcP. We were surprised to find that only 3 (14%) HIV-uninfected, exposed children developed PcP, compared with 11 (34%) of unexposed children. The reasons for this are unclear, but risk factors other than HIV infection may have played a role in the development of PcP. Case reports have suggested that HIV–uninfected exposed children are also at increased risk for the development of PcP or other lower respiratory infections [29,30,34].

 Some experimental studies have shown that Msg contains protective B and T cell antigenic determinants [36-38], but other reports using different models did not confirm these observations [55]. A recent prospective study we conducted of 550 adult HIV-infected and HIV-uninfected patients hospitalized with cough >2 weeks in Kampala, Uganda showed that lower serum IgM antibody responses to MsgC3 and MsgC8 were both associated with increased in-hospital mortality (48). In the current study, we found that increased IgG antibody levels to MsgC3 and IgM antibody levels to MsgC9 were associated with a reduction in PcP-related mortality. Taken together, the results of these two studies have led us to hypothesize that these antibodies are protective or contribute to the protection from severe PcP.

This study has several limitations. We did not determine whether the defect in PcP antibodies was limited to this organism or whether it was part of a broader global defect in antibody production associated with HIV infection. Further studies of the functional properties of these antibodies and of the serologic responses to other infectious agents are needed. Another limitation of this study is that it is a cross-sectional study. Longitudinal studies in HIV-infected children are needed to better understand the immune responses to this organism over time, and the factors that affect these responses. The present report only analyzed systemic antibody responses. Further analysis of local (respiratory tract) antibody responses and interactions between native Msg variants present in the cell wall of *P. jirovecii* would be of interest. Although we found RT-PCR to be sensitive and specific for PcP, further studies in other pediatric populations are needed. The small number of HIV-uninfected, exposed children with PcP precluded evaluation of the effects of HIV exposure on development of PcP. Additional investigation of larger numbers of these individuals would be very helpful.

In summary, this study provides new information about the humoral immune responses of young HIV-infected and HIV-uninfected children to *P. jirovecii* antigens, and suggests future areas for investigation. 
